# Optimization of a new selective pressurized liquid extraction methodology for determining organic pollutants in wild boar livers

**DOI:** 10.1016/j.mex.2021.101242

**Published:** 2021-01-23

**Authors:** Xiana González-Gómez, Noelia Cambeiro-Pérez, María Figueiredo-González, Elena Martínez-Carballo

**Affiliations:** Universidade de Vigo, Campus da Auga, Analytical and Food Chemistry Department, Facultade de Ciencias 32004, Ourense, Spain

**Keywords:** Organic pollutants, Wild boar livers, Selective pressurized liquid extraction, GC-QqQ-MS/MS

## Abstract

In this study, a new selective pressurised liquid extraction (SPLE) methodology was optimised for determining about 70 organic pollutants (OPs) including organochlorine (OCPs), organophosphate (OPPs) and pyrethroid (PYRs) pesticides, polychlorinated biphenyls (PCBs), polybromodiphenyl ethers (PBDEs), as well as, polycyclic aromatic hydrocarbons (PAHs) in wild boar liver samples considering the temperature, pressure and time of contact between the solvent and the matrix as influential variables. Clean-up of extracts was performed by solid-phase extraction (SPE) using EZ-POP cartridges. Detection of OPs was carried out by gas chromatography (GC) coupled to tandem mass spectrometry (QqQ-MS/MS). This new approach offers:

• A new non-time consuming SPLE methodology for determining about 70 OPs in wild boar.
• Recoveries achieved ranged between 74 to 119 % with RSD less than 20 %.
• Detection and quantification limits in the low to mid pg/g range.

## Graphical abstract

**Figure:**
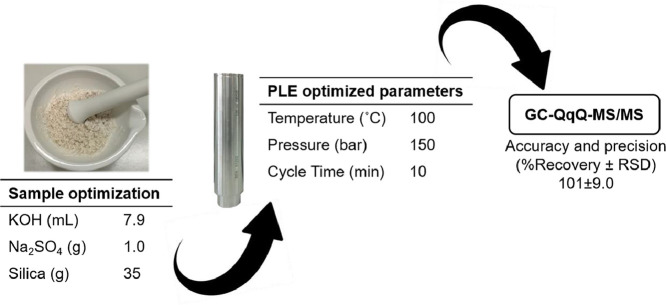

Specifications table

**Table:** 

Subject Area	Environmental Science
More specific subject area	Biomonitoring environmental pollutants
Method name	Selective pressurized liquid extraction
Name and reference of original method	X. González-Gómez, N. Cambeiro-Pérez, E. Martínez-Carballo, J. Simal-Gándara, Screening of organic pollutants in pet hair samples and the significance of environmental factors. Sci. Total Environ. 625 (2018) 311–319.
Resource availability	

It is well-known that living organisms are exposed to organic pollutants (OPs) release into the environment. These chemical inputs from different sources (industry, urban and agricultural areas) may create a vulnerable status especially to animals [1,2]. The great variety of OPs that could be responsible for this type of damage makes necessary the development of powerful analytical methodologies, which allow the identification of these substances.

## Reagents and standards

A list of the target OPs and the labelled internal and surrogate standards including CAS and supplier is given in Table S1 and S2, respectively. In order to improve peak shape and reduce OP decomposition, the 3-ethoxy-1,2-propanediol (98 %), d-sorbitol (> 99 %) and l-gulonic acid γ-lactone (> 98 %) used as analyte protectants (APs) were purchased from Sigma Aldrich (Madrid, Spain). Individual stock standard solutions of APs were prepared in acetonitrile (50 g/L), acetonitrile:water (85:15, v/v, 5 g/L) and acetonitrile:water (80:20, v/v, 5 g/L), respectively. Mixes of 10 mg/L stock solutions of each family of OPs were prepared from the individual stock solutions standards in acetonitrile. From these solutions, standards ranging from 0.25 to 100 μg/L were prepared in APs and used to construct the calibration line. These solutions were stored in amber flasks at - 18°C.

## SPLE optimisation

Liver is a complex matrix that requires a sample preparation in order to improve the in-situ clean-up in sample procedure. The effect of the presence or the absence of additives (KOH (aqueous) (60 %, w/v) or KOH (MeOH) (35 %, w/v)) used in other analytic techniques such as matrix solid phase dispersion (MSPD) were tested. Several combinations of KOH (aq) (30-60 %, w/v) and KOH (MeOH) (10-35 %, w/v) were chosen for such purpose. With the use of KOH (60 %, w/v) fat elimination was observed. Therefore, different combinations of mL KOH (15, 10 and 7.5) as well as activated silica amounts (35, 30 and 25 g) were tested. The experimental runs were performed in 2.0 g liver samples spiked with OP concentration range at 0.25 ng/g and 0.50 ng/g. Response was evaluated in terms of the recoveries of the selected OPs. Determination by GC-QqQ-MS/MS were performed using a previous ones optimised by the present research team [2] (Table S3).

**Fig. 1:**
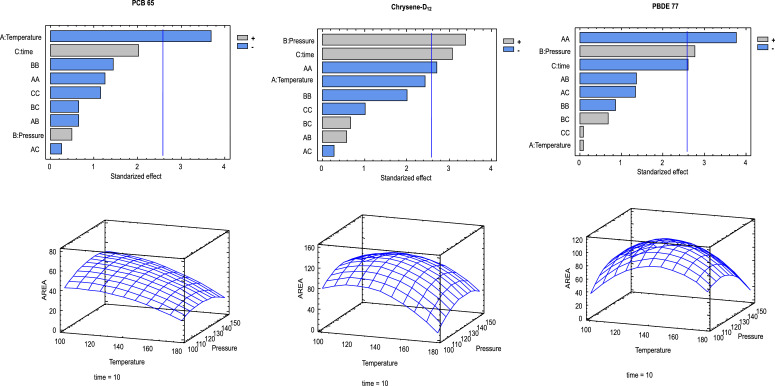
A) Pareto charts for main factors and two-factor interactions for the selected surrogates PCB65, chrysene-D_12_ and PBDE77, as well as response surface for 10 min cycle time. The single factors selected were: A (Temperature), B (pressure) and C (Cycle Time).

The optimal sample conditions were 7.5 mL KOH (60%, w/v), 35 g activated silica and 1.0 g of anhydrous sodium sulphate. To extract the maximum target analytes with minimum interferences, different SPLE parameters were optimized. The selected parameters were temperatures (100 ˚C, 137.5 ˚C and 175˚C), static times (5 min, 10 min and 15 min) and pressures (100 ba, 125 ba and 150 ba) using acetonitrile as solvent by a Box and Benhken experimental design with three independent variables consisted of 15 random experimental runs including three replicates at the central point. The experimental design was generated and all analytical treatments were supported by the software Statgraphics Plus 5.1 version (Manugistics, Rockville, MD, USA). The results are shown in Fig. 1. The final working conditions were obtained at 100 ˚C, three extraction cycles (10 min) and 150 ba.

Dual-layer EZ-POP SPE cartridges were used after SPLE. The final acetonitrile extract (1.0 mL) was passed through the tandem of dual-layer EZ-POP SPE cartridges previously conditioned with 20 mL of acetone. Acetonitrile (40 mL) was used to elute the target analytes and the collected extract was again reduced until dryness at 30°C, re-dissolved in 100 µL of acetone containing 50 ng of the internal standards and the three APs for GC-QqQ-MS/MS.

## Quality assurance/quality control (QA/QC)

The studied methods were in-house validated according to the criteria and recommendations of European guidelines for linearity, precision, trueness/accuracy, limits of detection and quantification (LODs and LOQs) and uncertainly values [3]. Internal linear calibration was used to quantify the targeted OPs in livers using the following internal standards: DDT-D_8_ for OCPs, diazinon-D_10_ for OPPs, PCB 30 and 195 for PCBs, *trans*-cypermethrin-D_6_ for PYRs and PBDE 166 for PBDEs. Linear calibration curves fit reasonably (r^2^ > 0.999) in a twelve-point calibration curve with a concentration scale of two or three orders of magnitude, depending on the compound (0.010 – 1.0 ng/g). The quality parameters of the optimised method are summarized in Table 2. Results obtained for the accuracy were in the range from 1.0 to 14 %.

For the validation of the analytical methodology, 29 liver samples from Ourense (Northwest of Spain) were analysed. The set of liver samples was processed each day together with: a reagent blank to test for contamination in the extraction process, a spiked blank and a spiked sample at an intermediate concentration (0.50 ng/g) to calculate the extraction efficiency. Surrogate standards (chlorpyrifos-D_10_, α,γ-HCH-D_6_; DDE-D_8_; HCB-^13^C_6_, Chrysene-D_12_, PBDE 77, PCB 14, 65 and 166, *trans*-Permethrin-D_6_) were also added to check the recovery rates in each extraction procedure.

**Fig. 2:**
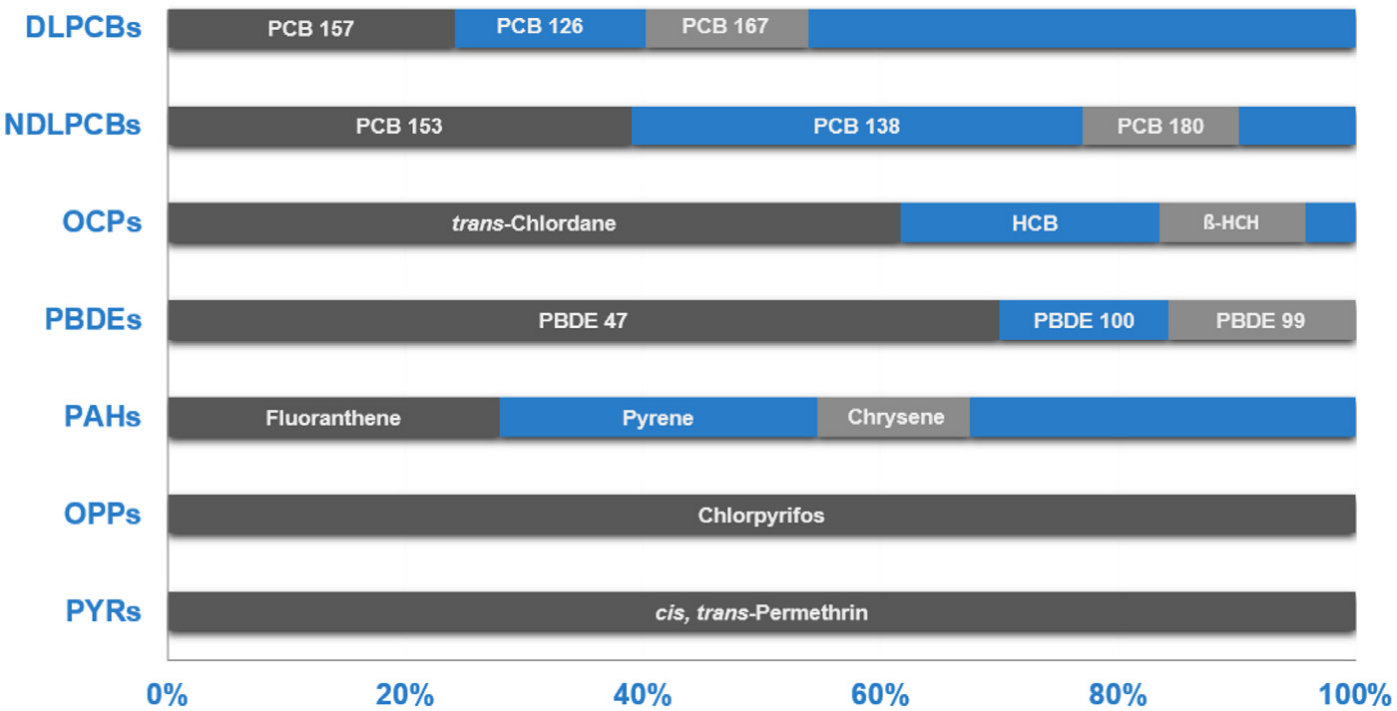
Summary of the main OP contributors in each family.

Most of the target pollutants were detected in the selected liver samples with significant differences (p < 0.050) and the following mean level concentration order ΣPAHs > ΣOCPs > ΣNDLPCBs > ΣPYRs > ΣOPPs > ΣDLPCBs > ΣPBDEs. Fig. 2 shows the main contributors in each family of OPs. Fluoranthene and pyrene were the main PAHs found in liver. With regard to chlorinated pollutants, *trans*-Chlordane were the most abundant OCP followed by HCB, as well as, PCB 153, 138 and 180 for NDLPCBs. PCB 157 and PCB 126 were the most prevalent DLPCB congeners. Permethrin and chlorpyrifos were the detected PYRs and OPPs, respectively. To our knowledge no results were found about the levels of PYR and OPP pesticides in liver of wild terrestrial mammals. PBDEs were the OPs with the lowest contribution with PBDE 47, 100 and 99 as major congeners.

## Additional information

To our knowledge, scarce literature about the concentration of OPs in wild boar liver is available due to the complexity of the selected biological sample [4], [5], [6], [7], [8], [9], [10], [11], [12], [13], [14], [15], [16], [17], [18], [19], [20]. For these reasons, it is required to develop quick and simple techniques capable of efficiently detecting a wide range of contaminants. In this type of multiresidue methods the extraction process is perhaps the most critical step since it requires the development of special and suitable conditions to determine substances with different physico-chemical properties related to water solubility (S_w_), octanol/water partition coefficient (K_ow_) and organic carbon partition coefficient (K_oc_). In recent years, OPs have been analysed in the liver of different wild animals (Table 1). Most of the studies focus on the determination of a single group of compounds. These researches use classical extractive techniques such as solid-liquid extraction (SLE) or soxhlet followed by clean-up steps using gel permeation chromatography (GPC) or solid-phase extraction (SPE with different absorbents (silica, alumina, florisil...) [[4], [5], [6],[8], [9], [10], [11], [12], [13], [14], [15], [16], [17]]. The main disadvantages of these techniques are the use of large amounts of solvent and the need for additional cleaning steps to avoid interferences, which involves possible loss of analytes and waste of time. Other alternatives are the use of high pressure extractive techniques such as accelerated solvent extraction (ASE) or also called pressurized liquid extraction (PLE) [7,[18], [19], [20], [21], [22]]. The combination of PLE with an in situ clean-up (in cell) of the extract is known as selective pressurized liquid extraction (SPLE). This technique avoids the need of subsequent cleanings and also improves the automation of the process. To the best of our knowledge, there is only a research in sheep liver where only PBDEs and PCBs were analysed by this technique [18]. Something new and promising is the inclusion of additives such as potassium hydroxide in SPLE to avoid the co-elution of unwanted matrix components allowing the extraction of about 70 OPs.

**Table 1:** Background of the analytical extraction methods for liver from wild mammals since 2001.

Compounds	Specie	Extraction	Clean Up	References
OCPs, PCBs	Wild boar	SLE	Silica gel	[4]
OPPs	Wild boar	SLE	SPE C18	[5]
OCPs, PCBs	Wild boar	SLE	Florisil	[6]
PCDDs/DFs, PCBs	Wild boar	ASE	Acidic silica, florisil	[7]
PBDEs	Wild boar	Soxhlet	Acidic silica gel	[8]
OCPs, PCBs	Wolf	SLE	Alumina	[9]
PAHs	Otter	Soxhlet	GPC, silica gel	[10]
OCPs, PCBs	Lynx	SLE	Sulfuric acid	[11]
PCBs, OH-PCBs	Seal	SLE	Silica gel	[12]
PAHs	Dolphin	Soxhlet	GPC	[13]
PBDEs	Otter	SLE	GPC	[14]
PBDEs	Otter	SLE	Florisil	[15]
PCDD/Fs, PCBs, PBDEs	Reindeer	SLE	Multilayer column	[16]
OCPs, PCBs	Mink	SLE	Florisil	[17]
PBDEs, PCBs	Sheep	SPLE	Acidic silica, sodium sulphate	[18]
PCBs	Racoon dog	ASE	GPC	[19]

**Table 2:** Mean recoveries (R) and relative standard deviations (RSD) at four spike levels (LOQs, 0.10, 0.25 and 0.50 ng/g), LOD (ng/g) and LOQ (ng/g) for each target compound are shown.

OPs	RT (min)	% R (RSD)	LODs	LOQs
α-HCH-D_6_	6.860	109 (10)	-	-
α-HCH	6.912	89 (16)	0.010	0.050
HCB-^13^C_6_	7.043	94 (18)	-	-
HCB	7.043	112 (16)	0.32	1.1
PCB 14	7.079	108 (10)	-	-
β-HCH	7.309	117 (9.1)	0.020	0.19
γ-HCH-D_6_	7.344	93 (17)	-	-
PCB 11	7.079	89 (9.0)	0.010	0.040
Diazinon	7.544	94 (1.0)	0.11	0.38
PCB 28	8.027	83 (7.2)	0.0010	0.0040
Parathion Methyl	8.654	106 (17)	0.10	0.33
Heptachlor	8.765	106 (18)	0.010	0.040
Aldrin	9.289	117 (4.0)	0.010	0.040
PCB 52	9.293	92 (5.0)	0.010	0.040
PCB 65	9.293	105 (14)	-	-
Chlorpyrifos-D_10_	9.387	88 (19)	-	-
Fenthion	9.433	77 (5.0)	0.010	0.040
Chlorpyrifos	9.476	106 (15)	0.040	0.14
Fluoranthene	10.454	104 (11)	0.030	0.10
*trans*-Chlordane	10.880	95 (1.0)	0.010	0.040
Pyrene	11.101	100 (10)	0.11	0.39
*cis*-Chlordane	11.267	99 (3.0)	0.010	0.040
DDE-D_8_	11.752	99 (17)	-	-
PCB 101	11.752	92 (2.3)	0.010	0.040
PCB 105	11.752	108 (9.0)	0.010	0.040
PCB 77	11.753	102 (1.0)	0.010	0.040
PCB 81	11.753	103 (11)	0.010	0.040
*o,p’*-DDT	12.045	94 (4.0)	0.050	0.16
Dieldrin	12.677	120 (8.0)	0.010	0.040
Endrin	12.677	119 (8.3)	0.010	0.040
PCB 114	12.750	94 (14)	0.010	0.040
PBDE 28	13.530	87 (11)	0.0010	0.0020
*p,p’*-DDT	12.924	119 (12)	0.010	0.040
*o,p*’-DDT	13.058	94 (18)	0.030	0.12
PCB 138	13.364	89 (3.1)	0.010	0.040
PBEB	13.628	115 (12)	0.010	0.040
PCB123	13.979	92 (10)	0.010	0.040
PCB 118	13.979	93 (11)	0.010	0.060
*p,p’*-DDT	14.054	86 (18)	0.0080	0.020
PCB 153	14.616	99 (1.0)	0.010	0.040
PCB 166	14.616	108 (12)	-	-
PCB 156	14.616	81 (16)	0.010	0.040
PCB126	14.611	115 (16)	0.010	0.040
Chrysene-D_12_	15.569	89 (12)	-	-
Chrysene	15.553	99 (8.0)	0.010	0.060
B[a]A	15.553	85 (5.4)	0.020	0.080
PCB 180	15.942	106 (13)	0.010	0.040
PCB 157	15.937	85 (18)	0.010	0.040
PCB 167	15.937	117 (4.0)	0.010	0.040
PBDE 47	16.316	74 (9.2)	0.0020	0.0070
PCB 169	16.764	102 (1.0)	0.010	0.040
PBDE 77	17.657	93 (1.1)	-	-
PCB 189	18.037	90 (18)	0.010	0.040
*trans*-Permethrin	18.786	99 (5.0)	0.12	0.42
*cis*-Permethrin	19.026	99 (6.0)	0.12	0.42
*trans*-Permethrin-D_6_	19.087	97 (9.0)	-	-
PBDE 99	19.100	99 (6.0)	0.0010	0.0040
B[k]F	19.785	110 (13)	0.030	0.12
B[b]F	19.785	100 (1.3)	0.0030	0.010
PBDE 100	19.959	87 (4.0)	0.030	0.12
Cyfluthrin	20.294	110 (14)	0.050	0.16
Cypermethrin	20.489	111 (10)	0.10	0.33
B[a]P	20.923	116 (11)	0.010	0.030
PCB 209	21.276	114 (1.0)	0.010	0.040
PBDE 153	22.111	87 (8.0)	0.030	0.12
PBDE 154	23.589	113 (1.4)	0.050	0.16
Deltamethrin	23.621	92 (14)	0.050	0.16
DB[ah]A	25.636	115 (11)	0.010	0.040
B[ghi]P	26.089	105 (9.0)	0.010	0.060
I[123cd]P	26.089	105 (7.4)	0.13	0.43

^a^LOD = 3 * sb/b

^b^LOQ = 10 * sb/b (sb= standard deviation of the intercept; b=slope of the calibration curve). They were then tested experimentally by spiking five replicates of blank samples at such levels. To calculate LODs and LOQs the obtained values of unfortified liver samples were multiplied by the enrichment factors and by the recoveries of the analytes. To verify the limits for real samples, signal-to-noise ratios for the analytes in extracts of liver samples in which concentrations were close to the calculated LOQs were determined.

PCBs; Bobcat; ASE; Silica gel; [20].

## Declaration of Competing Interests

The authors declare that they have no known competing financial interests or personal relationships that could have appeared to influence the work reported in this paper.

## Appendix Supplementary materials

Image, application 1
