# Supplementary material for: Optimization of a new selective pressurized liquid extraction methodology for determining organic pollutants in wild boar livers
Source: MethodsX. 2021 Jan 23;8:101242. doi: 10.1016/j.mex.2021.101242 (PMC8374156; doi:10.1016/j.mex.2021.101242)
Supplement: Supplementary file 1 [file mmc1.docx]

**Supplementary material *and/or* Additional information:**

**Table S1.** Target list including CAS and supplier.

| **Compound** | **Abbreviation** | **CAS** | **Supplier** |
| --- | --- | --- | --- |
| α-hexachlorocyclohexane | α-HCH | 319-84-6 | Sigma-Aldrich |
| β-hexachlorocyclohexane | β-HCH | 319-85-7 | Sigma-Aldrich |
| Aldrin |  | 309-00-2 | Restek |
| Benzo[a]anthracene | B[a]A | 56-55-3 | Sigma-Aldrich |
| Benzo[a]pyrene | B[a]P | 50-32-8 | Sigma-Aldrich |
| Benzo[b]fluoranthene | B[b]F | 205-99-2 | Sigma-Aldrich |
| Benzo[ghi]perylene | B[ghi]P | 191-24-2 | Sigma-Aldrich |
| Benzo[k]fluoranthene | B[k]F | 207-08-9 | Sigma-Aldrich |
| Chlordane (tech mix) |  | 12789-03-6 | AccuStandard |
| Chlorpyrifos |  | 2921-88-2 | Sigma-Aldrich |
| Chrysene | Chr | 218-01-9 | Sigma-Aldrich |
| Cypermethrin |  | 52315-07-8 | Analytical Standard Solutions |
| Cyfluthrin |  | 68359-37-5 | Analytical Standard Solutions |
| Dibenzo[a,h]anthracene | DB[ah]A | 53-70-3 | Sigma-Aldrich |
| Dichlorodiphenyltrichloroethane | DDT (mixture *p,p’* & *o,p’*) | 8017-34-3 | Sigma-Aldrich |
| Deltamethrin |  | 52918-63-5 | Analytical Standard Solutions |
| Diazinon |  | 33-41-5 | Analytical Standard Solutions |
| Dieldrin |  | 60-57-1 | Restek |
| Endrin |  | 72-20-8 | Restek |
| Fenthion |  | 55-38-9 | Sigma-Aldrich |
| Fluoranthene | F | 206-44-00 | Sigma-Aldrich |
| Hexachlorobenzene | HCB | 41411-63-6 | AccuStandard |
| Heptachlor |  | 76-44-8 | Dr. Ehrenstorfer |
| Indene[1,2,3-cd]pyrene | I[123cd]P | 193-38-5 | Sigma-Aldrich |
| Parathion methyl |  | 298-00-0 | Sigma-Aldrich |
| 2,4,4′-tribromodiphenyl ether | PBDE 28 | 41318-75-6 | AccuStandard |
| 2,2',4,4'-tetrabromodiphenyl ether | PBDE 47 | 5436-43-1 | AccuStandard |
| 2,2',4,4',5-pentabromodiphenyl ether | PBDE 99 | 60348-60-9 | AccuStandard |
| 2,2′,4,4′,6-pentabromodiphenyl ether | PBDE 100 | 189084-64-8 | AccuStandard |
| 2,2',4,4',5,5'-hexabromodiphenyl ether | PBDE 153 | 68631-49-2 | AccuStandard |
| 2,2',4,4',5,6'-hexabromodiphenyl ether | PBDE 154 | 207122-15-4 | AccuStandard |
| 3,3′-dichlorobiphenyl | PCB 11 | 2050-67-1 | AccuStandard |
| 2,4,4′-trichlorobiphenyl | PCB 28 | 7012-37-5 | Sigma-Aldrich |
| 2,2′,5,5′-tetrachlorobiphenyl | PCB 52 | 35693-99-3 | Sigma-Aldrich |
| 3,3′,4,4′-tetrachlorobiphenyl | PCB 77 | 32598-13-3 | CPAchem |
| 3,4,4',5-tetrachlorobiphenyl | PCB 81 | 70362-50-4 | CPAchem |
| 2,2’,4,5,5’-pentachlorobiphenyl | PCB 101 | 37680-73-2 | Sigma-Aldrich |
| 2,3,3’,4,4’-pentachlorobiphenyl | PCB 105 | 322259814-4 | CPAchem |
| 2,3,4,4’,5-pentachlorobiphenyl | PCB 114 | 74472-37-0 | CPAchem |
| 2,3’,4,4’,5-pentachlorobophenyl | PCB 118 | 31508-00-6 | CPAchem |
| 2’,3,4,4’,5-pentachlorobophenyl | PCB123 | 65510-44-3 | CPAchem |
| 3,3’,4,4’,5-pentachlorobophenyl | PCB126 | 57465-28-8 | CPAchem |
| 2,2’,3,4,4’,5-hexachlorobiphenyl | PCB 138 | 35065-28-2 | Sigma-Aldrich |
| 2,2’,4,4’,5,5’-hexachlorobiphenyl | PCB 153 | 35065-27-7 | Sigma-Aldrich |
| 2,3,3’,4,4’,5-hexachlorobiphenyl | PCB 156 | 38380-08-4 | CPAchem |
| 2,3,3’4,4’,5’-hexachlorobiphenyl | PCB 157 | 69782-90-7 | CPAchem |
| 2,3’,4,4’,5,5’-hexachlorobiphenyl | PCB 167 | 52663-72-6 | CPAchem |
| 3,3’,4,4’,5,5’-hexachlorobiphenyl | PCB 169 | 32774-16-6 | CPAchem |
| 2,2’,3,4,4’,5,5’-heptachlorobiphenyl | PCB 180 | 35065-29-3 | Sigma-Aldrich |
| 2,3,3’,4,4’,5,5’-heptachlorobiphenyl | PCB 189 | 39635-31-9 | CPAchem |
| 2,2’,3,3’,4,4’,5,5’-decachlorobiphenyl | PCB 209 | 2051-24-3 | Sigma-Aldrich |
| Permethrin |  | 52645-53-1 | Analytical Standard Solutions |
| Pyrene | P | 129-00-0 | Sigma-Aldrich |

**Table S2.** Labelled internal and surrogate standards including CAS and supplier.

| **Compound** | **Abbreviation** | **CAS** | **Type** | **Family** | **Supplier** |
| --- | --- | --- | --- | --- | --- |
| α-hexachlorocyclohexane-D_6_ | α-HCH-D_6_ | 86194-41-4 | Surrogate | OCs | Analytical Standard Solutions |
| γ–hexachlorocyclohexane-D_6_ | γ-HCH-D_6_ | 60556-82-3 | Surrogate |  | Analytical Standard Solutions |
| Hexachlorobenzene-^13^C_6_ | HCB-13C_6_ | 93952-14-8 | Surrogate |  | Analytical Standard Solutions |
| Chlorpyriphos-D_10_ |  | 285138-81-0 | Surrogate | OPPs | Analytical Standard Solutions |
| Chrysene-D_12_ | Chr-D_12_ | 1719-03-5 | Surrogate | PAHs | Sigma-Aldrich |
| 1,1-dichloro-2,2-bis(4-chlorophenyl-D4)ethylene | DDE-D_8_ | 93952-19-3 | Surrogate | DDTs | Analytical Standard Solutions |
| 1,1,1-trichloro-2,2-bis(4-chlorophenyl-D4)ethane | DDT-D_8_ | 93952-18-2 | Internal Standard |  | CDN Isotopes |
| 3,3′,4,4′-Tetrabromodiphenyl ether | PBDE 77 | 93703-48-1 | Surrogate | PBDEs | Sigma-Aldrich |
| 3,5-Dichlorobiphenyl | PCB14 | 34883-41-5 | Surrogate | PCBs | AccuStandard |
| 2,4,6-Trichlorobiphenyl | PCB 30 | 35693-92-6 | Internal Standard |  | AccuStandard |
| 2,3,5,6-Tetrachlorobiphenyl | PCB 65 | 33284-54-7 | Surrogate |  | AccuStandard |
| 2,3,4,4',5,6-Hexachlorobiphenyl | PCB 166 | 41411-63-6 | Surrogate |  | AccuStandard |
| *trans*-Cypermethrin-D_6_ | t-Cypermethrin-D_6_ | 82523-65-7 | Internal Standard | PYRs | Dr. Ehrenstorfer |
| *cis*-Permethrin-^13^C_6_ | c-Permethrin-^13^C_6_ | 61949-76-6 | Surrogate |  | Cambridge Isotope Laboratories |

**Table S3.** GC-QqQ-MS/MS detection parameters.

| **RT (min)** | **Compound** | **MRM quantification** | **Dwell (ms)** | **CE (eV)** | **MRM confirmation** | **Dwell (ms)** | **CE (eV)** |
| --- | --- | --- | --- | --- | --- | --- | --- |
| 6.860 | α-HCH-D_6_ | 224 > 152 | 15 | 30 | 224 > 189 | 20 | 6.0 |
| 6.912 | α-HCH | 217 > 181 | 15 | 10 | 181 > 109 | 15 | 30 |
| 7.043 | HCB-^13^C_6_ | 290 > 255 | 10 | 30 | 191 > 257 | 25 | 20 |
| 7.043 | HCB | 284 > 214 | 10 | 30 | 284 > 249 | 15 | 30 |
| 7.079 | PCB 14 | 222 > 152 | 15 | 30 | 224 > 152 | 15 | 30 |
| 7.134 | PCB 30 | 256 > 186 | 10 | 25 | 256 > 151 | 10 | 50 |
| 7.309 | β-HCH | 217 > 181 | 15 | 10 | 181 > 109 | 15 | 30 |
| 7.344 | γ-HCH-D_6_ | 224 > 152 | 15 | 30 | 224 > 189 | 20 | 6.0 |
| 7.079 | PCB 11 | 222 > 152 | 15 | 30 | 224 > 152 | 15 | 30 |
| 7.544 | Diazinon | 304 > 179 | 15 | 10 | 199 > 135 | 15 | 10 |
| 8.027 | PCB 28 | 256 > 186 | 10 | 25 | 256 > 151 | 10 | 50 |
| 8.654 | Parathion methyl | 263 > 109 | 15 | 10 | 125 > 79 | 15 | 10 |
| 8.765 | Heptachlor | 271 > 237 | 50 | 16 | 274 > 239 | 50 | 16 |
| 9.289 | Aldrin | 255 > 220 | 10 | 20 | 263 > 193 | 15 | 40 |
| 9.293 | PCB 52 | 292 > 220 | 15 | 30 | 292 > 257 | 10 | 10 |
| 9.293 | PCB 65 | 292 > 220 | 15 | 30 | 292 > 257 | 10 | 10 |
| 9.387 | Chlorpyrifos-D_10_ | 324 > 260 | 20 | 10 | 324 > 292 | 20 | 10 |
| 9.433 | Fenthion | 278 > 109 | 10 | 30 | 279 > 169 | 10 | 15 |
| 9.476 | Chlorpyrifos | 314 > 258 | 15 | 10 | 314 > 286 | 10 | 5.0 |
| 10.454 | F | 202 > 152 | 10 | 42 | 202 > 201 | 10 | 27 |
| 10.880 | t-Chlordane | 373 > 266 | 15 | 30 | 373 > 301 | 10 | 10 |
| 11.101 | P | 202 > 151 | 10 | 42 | 202 > 201 | 10 | 27 |
| 11.267 | c-Chlordane | 373 > 266 | 15 | 30 | 373 > 301 | 10 | 10 |
| 11.752 | DDE-D_8_ | 326 > 254 | 10 | 20 | 326 > 35 | 10 | 20 |
| 11.752 | PCB 101 | 326 > 256 | 10 | 30 | 254 > 184 | 15 | 40 |
| 11.752 | PCB 105 | 326 > 256 | 15 | 30 | 373 > 301 | 10 | 10 |
| 11.753 | PCB 77 | 292 > 220 | 15 | 30 | 292 > 257 | 10 | 10 |
| 11.753 | PCB 81 | 292 > 220 | 15 | 30 | 292 > 257 | 10 | 10 |
| 12.045 | *o,p’*-DDT | 235 > 165 | 15 | 20 | 235 > 199 | 15 | 20 |
| 12.677 | Dieldrin | 277 > 241 | 10 | 5.0 | 263 > 228 | 10 | 20 |
| 12.677 | Endrin | 263 > 193 | 15 | 40 | 281 > 173 | 15 | 40 |
| 12.750 | PCB 114 | 326 > 256 | 10 | 30 | 254 > 184 | 15 | 40 |
| 13.530 | PBDE 28 | 406 > 246 | 15 | 27 | 408 > 248 | 10 | 20 |
| 12.924 | *p,p’*-DDT | 235 > 165 | 15 | 20 | 235 > 199 | 15 | 20 |
| 13.058 | *o,p’*-DDT | 235 > 165 | 15 | 20 | 235 > 199 | 15 | 20 |
| 13.364 | PCB 138 | 360 > 290 | 15 | 40 | 360 > 235 | 10 | 15 |
| 13.628 | PBEB | 500 > 485 | 15 | 30 | 500 > 340 | 15 | 30 |
| 13.979 | PCB123 | 326 > 256 | 10 | 30 | 254 > 184 | 15 | 40 |
| 13.979 | PCB 118 | 326 > 256 | 10 | 30 | 254 > 184 | 15 | 40 |
| 14.054 | *p,p’*-DDT | 235 > 165 | 15 | 20 | 235 > 199 | 15 | 20 |
| 14.100 | DDT-D_8_ | 243 > 172 | 25 | 30 | 326 > 154 | 10 | 30 |
| 14.616 | PCB 153 | 360 > 290 | 15 | 40 | 360 > 325 | 10 | 15 |
| 14.616 | PCB 166 | 360 > 290 | 15 | 40 | 360 > 325 | 10 | 15 |
| 14.616 | PCB 156 | 360 > 290 | 15 | 40 | 360 > 325 | 10 | 15 |
| 14.611 | PCB126 | 326 > 256 | 10 | 30 | 254 > 184 | 15 | 40 |
| 15.569 | Chr-D_12_ | 240 > 236 | 10 | 35 | 240 > 238 | 10 | 35 |
| 15.553 | Chr | 228 > 226 | 10 | 40 | 228 > 202 | 10 | 35 |
| 15.553 | B[a]A | 228 > 226 | 10 | 40 | 228 > 202 | 10 | 35 |
| 15.942 | PCB 180 | 394 > 324 | 15 | 30 | 394 > 359 | 10 | 15 |
| 15.937 | PCB 157 | 360 > 290 | 15 | 40 | 360 > 325 | 10 | 15 |
| 15.937 | PCB 167 | 360 > 290 | 15 | 40 | 360 > 325 | 10 | 15 |
| 16.316 | PBDE 47 | 486 > 326 | 15 | 27 | 484 > 326 | 15 | 27 |
| 16.764 | PCB 169 | 360 > 290 | 15 | 40 | 360 > 325 | 10 | 15 |
| 17.657 | PBDE 77 | 486 > 326 | 15 | 27 | 484 > 326 | 15 | 27 |
| 18.037 | PCB 189 | 394 > 324 | 15 | 30 | 394 > 359 | 10 | 15 |
| 18.776 | c-Permethrin-^13^C_6_ | 189 > 159 | 10 | 17 | 189 > 171 | 10 | 17 |
| 18.786 | t-Permethrin | 183 > 168 | 15 | 17 | 183 > 153 | 15 | 17 |
| 19.026 | c-Permethrin | 183 > 168 | 15 | 17 | 183 > 153 | 15 | 17 |
| 19.100 | PBDE 99 | 566 > 406 | 15 | 27 | 406 > 297 | 10 | 32 |
| 19.785 | B[k]F | 252 > 250 | 10 | 45 | 252 > 224 | 10 | 60 |
| 19.785 | B[b]F | 252 > 250 | 10 | 45 | 252 > 224 | 10 | 60 |
| 19.959 | PBDE 100 | 566 > 406 | 15 | 27 | 406 > 297 | 10 | 32 |
| 20.195 | t-Cypemethrin-D_6_ | 169 > 133 | 15 | 5.0 | 171 > 135 | 15 | 5.0 |
| 20.294 | Cyfluthrin | 163 > 127 | 15 | 7.0 | 226 > 206 | 10 | 17 |
| 20.489 | Cypermethrin | 181 > 152 | 15 | 27 | 181 > 127 | 10 | 37 |
| 20.923 | B[a]P | 252 > 250 | 15 | 27 | 252 > 224 | 10 | 60 |
| 21.276 | PCB 209 | 498 > 428 | 15 | 30 | 498 > 426 | 15 | 30 |
| 22.111 | PBDE 153 | 644 > 484 | 10 | 27 | 484 > 375 | 10 | 37 |
| 23.589 | PBDE 154 | 643 > 484 | 10 | 27 | 484 > 375 | 10 | 37 |
| 23.621 | Deltamethrin | 253 > 174 | 10 | 7.0 | 251 > 172 | 10 | 7.0 |
| 25.636 | DB[ah]A | 278 > 276 | 10 | 42 | 278 > 250 | 10 | 60 |
| 26.089 | B[ghi]P | 276 > 274 | 10 | 50 | 276 > 272 | 10 | 60 |
| 26.089 | I[123cd]P | 276 > 274 | 10 | 50 | 276 > 272 | 10 | 60 |
